# To Dab or Not to Dab: Rising Concerns Regarding the Toxicity of Cannabis Concentrates

**DOI:** 10.7759/cureus.1676

**Published:** 2017-09-11

**Authors:** Saeed K Alzghari, Victor Fung, Shannon S Rickner, Liza Chacko, Steven W Fleming

**Affiliations:** 1 Gulfstream Genomics, Gulfstream Diagnostics; 2 Department of Emergency Medicine, Parkland Health and Hospital System, Dallas Texas; 3 Department of Emergency Medicine, UT Southwestern Medical Center; 4 Reference Health Laboratories, Gulfstream Diagnostics

**Keywords:** marijuana, dabs, dabbing, cardiotoxicity, psychosis, neurotoxicity, cannabis, cannabis concentrates

## Abstract

Cannabis use is steadily rising in the United States. As the popularity of marijuana rises, new varieties of cannabis-related products are becoming available. Dabs are cannabis concentrates gaining notoriety for their significant amounts of tetrahydrocannabinol (THC) that are ultimately vaporized and inhaled for their effect. Herein, we provide an overview of recent cases of dabbing to bring awareness to the clinicians, of the significant adverse effects associated with dabs including psychosis, neurotoxicity, and cardiotoxicity.

## Editorial

The popularity of cannabis has been growing in recent decades. Among adults in the United States, this rise in the use of cannabis can be attributed to more states deciding to legalize marijuana [1]. Currently, 29 states have legalized medical marijuana, eight of these states have recreational marijuana laws and likely, both numbers will continue to rise [2].

**Figure 1:**
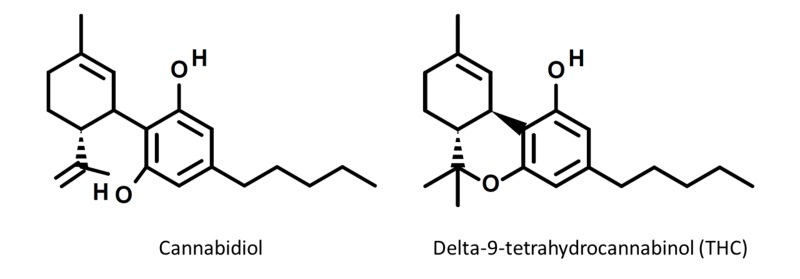
Structures of cannabidiol and delta-nine-tetrahydrocannabinol (THC).

Since the number of people using cannabis for recreational purposes is increasing, there are varieties of cannabis preparations that are becoming more available to the public. Some of the varieties of cannabis concentrates include “oil,” “budder,” "crumble," “wax,” and “dabs” that are typically created by taking Butane solvent and extracting the cannabinoid for a more potent product [3]. A method of consumption that is gaining notoriety is dabbing. Dabbing is defined as “a new method of consumption of cannabis whereby a cannabis concentrate is volatilized via application to a hot platform and the vapor is subsequently passed through a water pipe device and inhaled by the end user” [3]. The two main compounds typically found in dabs are cannabidiol (CBD) and tetrahydrocannabinol (THC) (Figure 1). Recently published case reports have shown significant psychosis, neurotoxicity, and cardiotoxicity associated with dabs [4-5]. In these three cases presented, each subject was a male in their teens or twenties and used some form of dab via inhalation (Table 1). None of the cases reported the amount or the dose of the dabs taken leading to toxicity. Two of the subjects presented with paranoia-like symptoms and one subject presented with seizure-like activity. Two of the subjects reported marked hypertension along with fever upon presentation. One case noted elevated troponins signifying cardiotoxicity in the form of myocardial injury [4]. The treatment using a benzodiazepine occurred in two cases. Furthermore, the treatment using an antipsychotic occurred in two of the cases. Of note, Rickner and colleagues analyzed the dab sample which the patient was using by mass spectrometry, revealed that the level of THC was 20.5% by weight and without any detectable level of cannabidiol (CBD) [4].

**Table 1:** Recent case reports involving the use of “dabs” and their outcomes. Abbreviations: h: hour, HD: hospital day, kg: kilograms, LCMS: liquid chromatography-mass spectrometry, mcg: micrograms, mg: milligrams, mmHg: millimeters of mercury, ng/mL: nanograms per milliliter, THC: tetrahydrocannabinol, TID: three times per day.

Author	Date	Country	Age (Years)	Sex	Drug(s)	Route	Urine THC Level	Presentation	Treatment and Outcome
Rickne, et al. [4]	June 2017	USA	17	M	Dabs; e-cigarette	Inhalation	carboxy-THC: 108 ng/mL (detection limit 15 ng/mL)	Seizure-like activity; alert but agitated; hyperthermic; hypertensive with systolic blood pressures in the 190s; twitches and jerks of his extremities; elevated troponins; liquid chromatography-mass spectrometry (LCMS) of dab sample contained THC at 20.5% and cannabidiol (CBD) was undetectable	Received propofol infusion (75 mcg/kg/min) followed by midazolam (2.5 mg/h) and fentanyl (125 mcg/h) for sedation; patient discharged without neurologic sequelae after five-day hospital stay
Pierre, et al. [5]	Feb 2016	USA	17	M	Recreational cannabis; cannabis wax	Inhalation	N/A	Over the course of three weeks after using cannabis wax, experienced paranoia; appeared confused, disorganized, and agitated; mild fever; tachycardia (up to 110 beats/minute); hypertension (up to 170/90 mmHg); diaphoresis; photophobia	Received risperidone 3 mg/day over one week where he returned to baseline mental status after 12-day hospital stay
Pierre, et al. [5]	Feb 2016	USA	26	M	Medical marijuana; cannabis wax (“Fire OG” and “Mystery”)	Inhalation	N/A	Over the course of 18 months, the patient became increasingly restless, confused, and disorganized; signs of paranoia	Treated with olanzapine 20 mg/day on HD one, transitioned to risperidone 2 mg/day on HD two, risperidone discontinued on HD three with little improvement; by HD seven and eight, patient experienced catatonia resulting in initiation of lorazepam 2 mg three times per day(TID) that resulted in resolution of catatonia followed by restarting risperidone 4 mg/day; discharged after 17-day hospital stay

Dabs that are homemade or obtained from an unreliable source may be prone to containing residual solvent (i.e. Butane, pesticides, or other contaminants). Raber and colleagues performed a study analyzing 57 samples of cannabis concentrates for contaminants and found that over 80% of the samples were contaminated by solvents or pesticides in some form [3]. Another interesting finding was that 56 out of the 57 samples had maximum THC concentrations ranging from 23.7% to 75.9%. One could argue that contaminants might play a role in the toxicity experienced in the cases presented; however, the dab sample analyzed by Rickner and colleagues revealed that concentrated levels of THC might play a bigger role in regard to toxicity [4]. There is a need for more research on the toxic dose of THC in humans in order to guide the clinicians in treating the patients experiencing adverse effects associated with dabs and other cannabis-related products.

In conclusion, medical providers need to be more aware of the dangers of dabbing. Dabs can have varying levels of contaminants, THC and CBD levels that could potentially trigger a toxidrome leading to psychosis, neurotoxicity, or cardiotoxicity. We advocate for more research into how the concentration of THC in dabs can lead to such adverse effects.
